# Dorsal penile nerve block with ropivacaine versus intravenous tramadol for the prevention of catheter-related bladder discomfort: study protocol for a randomized controlled trial

**DOI:** 10.1186/s13063-015-1130-2

**Published:** 2015-12-30

**Authors:** Jing-yi Li, Ren Liao

**Affiliations:** Department of Dermatovenereology, West China Hospital of Sichuan University, 37 Guoxue Lane, Chengdu, 610041 Sichuan Province People’s Republic of China; Department of Anesthesiology, West China Hospital of Sichuan University, 37 Guoxue Lane, Chengdu, 610041 Sichuan Province People’s Republic of China

**Keywords:** Randomized controlled trial, Dorsal penile nerve block, Catheter-related bladder discomfort

## Abstract

**Background:**

Catheter-related bladder discomfort (CRBD) is common in male patients under general anesthesia, and it may cause patient agitation and exacerbated postoperative pain. In this study, we will enroll male patients undergoing elective surgery with urinary catheterization after anesthetic induction and compare the efficacy of a dorsal penile nerve block (DPNB) and intravenous tramadol for the prevention of CRBD.

**Methods/Design:**

This trial is a prospective, open-label, randomized controlled trial that will test the superiority of a dorsal penile nerve block with 0.33 % ropivacaine to the use of intravenous tramadol 1.5 mg/kg for CRBD prevention. A total of 60 male patients undergoing elective surgery with urinary catheterization after anesthetic induction will be randomized to receive either DPNB with 0.33 % ropivacaine (DPNB group) or intravenous tramadol 1.5 mg/kg (TRAM group) after the completion of surgery but before extubation. The primary outcome is the incidence and severity of CRBD. Secondary outcomes include Visual Analog Score (VAS) for postoperative pain, number of patients requiring sulfentanil after operation, acceptance of an indwelling urinary catheter after extraction of the catheter, and postoperative side effects, which include postoperative nausea/vomiting (PONV), vertigo, sedation, drowsiness, and dry mouth.

**Discussion:**

For CRBD prevention, this trial is planned to test the superiority of a dorsal penile nerve block with 0.33 % ropivacaine to the use of intravenous tramadol 1.5 mg/kg. The results will provide new insight into the mechanism of CRBD and new clinical practice for the prevention of CRBD.

**Trial registration:**

The registration number is NCT01721031, which was assigned by the National Institutes of Health Clinical Trials Registry (ClinicalTrials.gov) on 27 October 27.

## Background

Catheter-related bladder discomfort (CRBD) due to an indwelling urinary catheter includes a distressing complex of symptoms such as an urge to void, irritation of the urethra, and discomfort in the supra-pubic region [1]. The incidence of CRBD ranges from 47 to 90 % in the postoperative period, especially in male patients who have had urinary catheterization after anesthetic induction under general anesthesia [1–4]. CRBD often causes patient agitation and is accompanied by behavioral responses that may include loud complaining and attempts to remove the urinary catheter. In addition, CRBD may result in exacerbated postoperative pain, increased postoperative complications, and prolonged hospital stay. Therefore, it is necessary to prevent and treat CRBD as early as possible.

The mechanism of CRBD has been reported to be similar to that of overactive bladder, that is, the involuntary contraction of the detrusor muscles of the urinary bladder, which is caused by acetylcholine released from activated cholinergic nerves and mediated by muscarinic receptors [1]. Therefore, muscarinic receptor antagonists such as tolterodine, oxybutynin, ketamine, butylscopolamine, and tramadol have been reported to be effective in the prevention or treatment of CRBD with a variable successful rate [5–9]. However, side effects such as sedation, nausea or vomiting, dry mouth, or other unpleasant complications may occur with these systemic administrations. Clinically, the dorsal penile nerve block (DPNB) has been applied in penile surgery for decades, and satisfactory pain relief effect could be achieved during operation and postoperatively without the side effects related to systemic administrations [10–13]. In addition, we have observed that patients undergoing urethral surgery with a urinary catheter left in-situ seldom complained about CRBD if the DPNB is performed at the end of operation. Based on these literatures and clinical practice, we hypothesize that DPNB could relieve CRBD for male patient with an indwelling urinary catheter if the insertion occurs after the induction of general anesthesia.

In this trial, we will compare the efficacy of a dorsal penile nerve block with 0.33 % ropivacaine to intravenous tramadol 1.5 mg/kg in the prevention of CRBD, as well as the incidences of postoperative side effects. To the best of our knowledge, this is the first trial that examines the use of the nerve block technique for CRBD prevention.

## Methods/Design

### Design

**Fig. 1:**
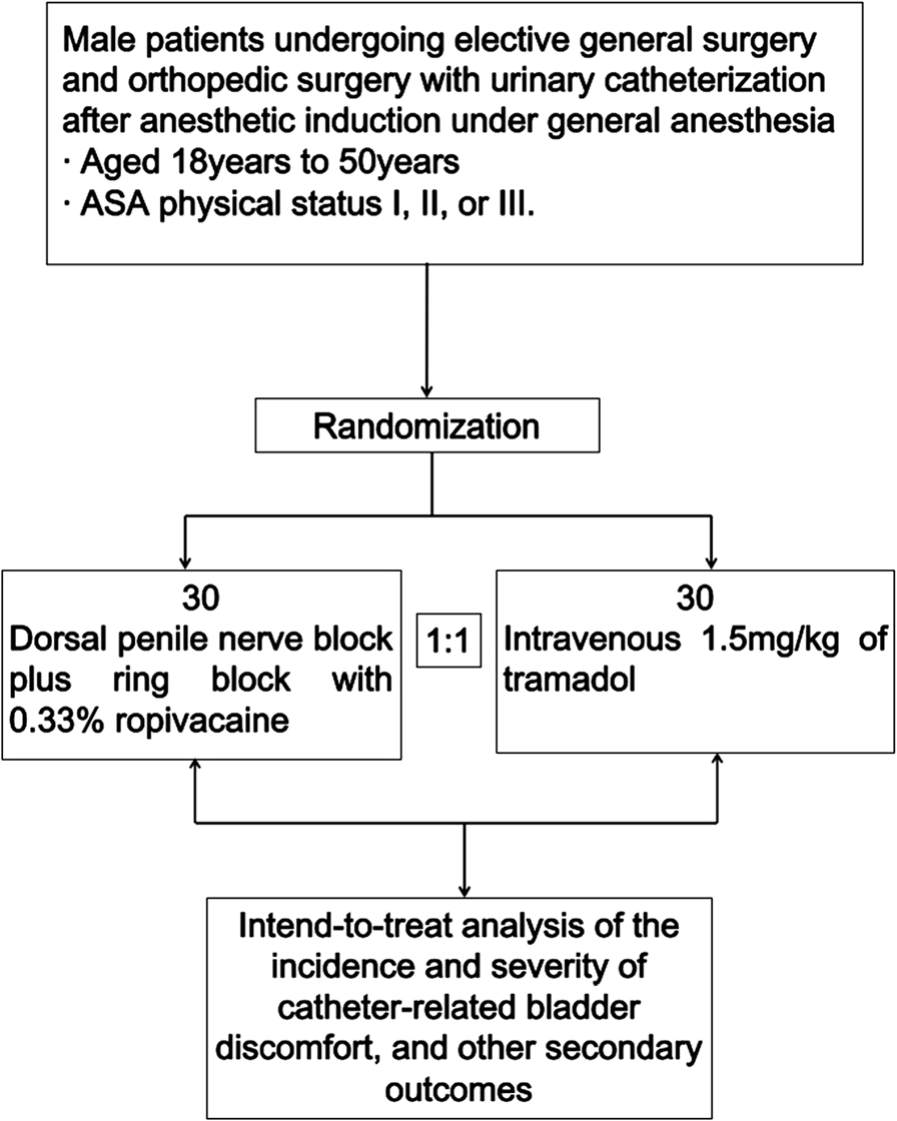
The dorsal penile nerve block (DPNB) trial flowchart

The DPNB trial is a prospective, open-label, randomized controlled trial that will test the superiority of a dorsal penile nerve block with 0.33 % ropivacaine to intravenous tramadol 1.5 mg/kg for CRBD prevention. The protocol of the trial has been registered at http://www.clinicaltrials.gov/NCT01721031 (NCT01721031), and a brief flowchart of the whole study is summarized in Fig. 1.

This trial is investigator initiated without any external financial sponsorship, and no company has played any role in the development, implementation, or data collection and analysis of this study. The authors and their colleagues are responsible for the trial design and execution; related statistical analysis; and all aspects of the manuscript preparation, including drafting, editing, and decisions on the final content. This study will be conducted according to the principles outlined in the Declaration of Helsinki. All investigators are appropriately qualified by training to conduct the trial. All patients have to sign the informed consent prior to study entry.

### Ethics

This study protocol has been approved by the Biological-Medical Ethical Committee of West China Hospital, Sichuan University, Chengdu, Sichuan, China, on 10 October 2012. Details of the study will be explained thoroughly to the potential subjects and/or his legal guardian by the investigator, and the signed informed consent form must be provided by all eligible patients before their participation in the study. Patients will be given at least 24 hours to decide whether they want to participate in the study. Participation in the study is entirely voluntary, and patients can withdraw from the study at any time. The privacy of all participants will be protected. Personal medical records will be checked by investigators and inspectors, and they will not export any confidential information. Data anonymity is ensured in the whole process of data management, and the data of all subjects will be kept within the Department of Anesthesiology, West China Hospital of Sichuan University.

### Recruitment

A total of 60 patients undergoing elective general surgery or orthopedic surgery with urinary catheterization (18 G Fr Foley catheter) after anesthetic induction under general anesthesia will be enrolled at two hospitals in China: the West China Hospital of Sichuan University and the First People’s Hospital of Neijang.

### Randomization and blinding

All patients recruited to the study will be collectively randomized using Statistical Analysis System (SAS 9.1) by the Department of Anesthesia, West China Hospital of Sichuan University. The responsible anesthesiologist will call the research nurse to get the randomization number and the allocated group. The investigators, responsible anesthesiologists, surgeons, and the research nurse will be aware of the treatment allocation. The patients, staff responsible for follow-up, and statisticians will be blind to the treatment assignment.

### Entry criteria

#### Inclusion criteria

The inclusion criteria are as follows:

1. Male patients aged 18 years to 50 years.
2. American Society of Anesthesiologists (ASA) physical status I, II, or III.
3. Undergoing elective general liver surgery and orthopedic extremity surgery with urinary catheterization after anesthetic induction under general anesthesia.
4. Glasgow Coma Scale (GCS) score of 15.
5. Ability to communicate.

#### Exclusion criteria

The exclusion criteria include the following:

1. Refusal to sign consent.
2. History of bladder dysfunction, such as overactive bladder (OAB, urinary frequency is more than three times in the night or more than eight times in 24 h).
3. Psychopathy.
4. Impaired renal function.
5. History of bladder outflow obstruction.
6. Neurogenic bladder.
7. Prostate disease.
8. Known allergies to any anesthetic agent.
9. Family history of malignant hyperthermia.
10. Coagulopathy.
11. Impairment of communication or cognition.
12. Active participation in another trial where the primary endpoint follow-up is ongoing.
13. Unwillingness or inability to comply with protocol procedures.

### Interventions

Patients in the DPNB group will be given a dorsal penile nerve block with 15 ml of 0.33 % ropivacaine after the completion of the surgery before extubation.

Patients in the TRAM group will be given tramadol of 1.5 mg/kg after the completion of surgery but before extubation.

### Outcome measures

All patients will be evaluated for outcomes at 0, 1, 2, 4, and 6 h after patients’ arrival in the post-anesthesia care unit (PACU) and after extraction of urinary catheter.

#### Primary outcome

The primary outcome is the incidence and severity of CRBD, which will be assessed according to the following scaling system: no CRBD indicates that there are no complaints of CRBD at all; mild indicates that complaints of CRBD exist only if the patient was asked about it; moderate indicates that patients complain of CRBD spontaneously; and severe indicates that the CRBD causes a spontaneous behavioral response such as flailing limb, strong vocal response, or attempt to pull out the catheter [2].

#### Secondary outcomes

The secondary outcomes include the following:

2.7.2.1. Visual Analogue Score (VAS) for postoperative pain.
2.7.2.2. Number of patients requiring postoperative sulfentanil.
2.7.2.3. Postoperative sufentanil requirement.
2.7.2.4. Duration of postoperative sulfentanil requirement.
2.7.2.5. Postoperative side effects, which include the following: (1) postoperative nausea/vomiting (PONV), (2) vertigo, (3) sedation, (4) drowsiness, and (5) dry mouth.
2.7.2.6. Acceptance of an indwelling urinary catheter after extraction of the catheter.

### Quality control

The principal investigator, study coordinator, and Office of Scientific Research at West China Hospital are jointly responsible for all aspects of the study protocol and amendments. Dr. Ren Liao, Associate Professor in the Department of Anesthesiology, West China Hospital, will be responsible for site monitoring. Data collection and follow-up will be performed by two dedicated affiliated research nurses. Designated trial monitors will periodically review all investigational data for accuracy and completeness to ensure protocol compliance.

### Sample size calculation

A difference of 30 % in the incidence of CRBD values between the DPNB group and the TRAM group is considered clinically important. Assuming a type I error protection of 0.05 and a power of 0.80, 24 patients in each group are required for a comparison within the group. Considering the difference between the two treatment groups at a 5 % significance level with an estimated 20 % dropout rate, 30 patients in each group are required in this study.

### Statistical analysis

All primary and secondary endpoints will be analyzed on an intent-to-treat basis. Analyses will be performed with the use of SPSS 18.0 software. The Student-t test will be used to analyze the demographic data in the two groups. The incidence of the CRBD and the side effects between the groups are analyzed by Chi-square test, whereas the severity of the CRBD (mild, moderate, and severe) will be analyzed by Fisher’s exact test. The VAS scale will be analyzed by the Mann–Whitney test. Postoperative sulfentanil requirement will be analyzed by *Z* test, and the duration of postoperative sulfentanil requirement and the number of patients requiring sulfentanil for postoperative pain will be analyzed with Fisher’s exact test. A *P* value < 0.05 is considered significant.

## Discussion

Tramadol is a weak opioid that is commonly used for postoperative pain relief [14, 15], and it is reported to halve the incidence of CRBD if it is administered i.v. 30 min before the end of surgery [9]. Therefore, we chose tramadol treatment as the control and are testing the superiority of a dorsal penile nerve block with 0.33 % ropivacaine over the intravenous tramadol 1.5 mg/kg in CRBD prevention.

Dorsal penile nerve block, a local anesthetic technique, has been applied widely in patients undergoing urethra surgery, and these patients, with a urinary catheter left in situ, rarely complain about CRBD. Theoretically, the nerve block should be associated with fewer side effects when compared with intravenous administration. Therefore, we hypothesize that DPNB could reduce the incidence of CRBD as well as the side effects. In addition, the afferent nerves of the urethra and bladder triangle are derived from sacral somatic (S2 to 4) [16], and a successful prevention of CRBD could be achieved by blocking the pudendal nerve. If the prevention of the CRBD can be achieved by the DPNB, a new mechanism of CRBD other than overactive bladder would be suggested by this trial.

In terms of the study design, the diameter of the 18 G Fr Foley catheter and the sex (male) has been reported to be independent predictors of moderate or severe CRBD in the PACU [1], so we choose male patients with 18 G Fr Foley catheter to test the effects of CRBD prevention in the current study. In addition, the local anesthetics are not applied for catheterization to minimize other factors related to CRBD. Furthermore, although we test the superiority of the dorsal penile nerve block with 0.33 % ropivacaine to intravenous tramadol 1.5 mg/kg in CRBD prevention, we are not going to cast doubt on the beneficial effects of anti-cholinergic drugs to relieve CRBD.

The limitation of this trial is that DPNB is only applicable to surgical patients undergoing general anesthesia. The application of DPNB should be studied in the future to benefit those patients who are undergoing catheterization for medical procedures not requiring any surgical intervention.

In conclusion, this trial plans to test the superiority of a dorsal penile nerve block with 0.33 % ropivacaine to intravenous tramadol 1.5 mg/kg in CRBD prevention. This study may provide a new insight into the mechanism of CRBD and new clinical practice for the prevention of CRBD.

## Trial status

The study is currently ongoing.

## Abbreviations

ASA: American Society of Anesthesiologists
CRBD: catheter-related bladder discomfort
DPNB: dorsal penile nerve block
GCS: Glasgow Coma Scale
OAB: overactive bladder
PACU: post-anesthesia care unit
PONV: postoperative nausea/vomiting
SAS: statistical analysis system
VAS: visual analog score
